# Geography, private costs and uptake of screening for abdominal aortic aneurysm in a remote rural area

**DOI:** 10.1186/1471-2458-6-80

**Published:** 2006-03-29

**Authors:** Sandra M Lindsay, John L Duncan, John Cairns, David J Godden

**Affiliations:** 1 Centre for Rural Health, University of Aberdeen, Beechwood Business Park, Inverness IV2 3BL, UK; 2 Department of Surgery, Raigmore Hospital, Inverness, IV2 3UJ, UK; 3 Health Economics Research Unit, University of Aberdeen, Polwarth Building Aberdeen, AB25 2ZD, UK

## Abstract

**Background:**

The relationship between geographical location, private costs, health provider costs and uptake of health screening is unclear. This paper examines these relationships in a screening programme for abdominal aortic aneurysm in the Highlands and Western Isles of Scotland, a rural and remote area of over 10,000 square miles.

**Methods:**

Men aged 65–74 (n = 9323) were invited to attend screening at 51 locations in 50 settlements. Effects of geography, deprivation and age on uptake were examined. Among 8,355 attendees, 8,292 completed a questionnaire detailing mode of travel and costs incurred, time travelled, whether accompanied, whether dependants were cared for, and what they would have been doing if not attending screening, thus allowing private costs to be calculated. Health provider (NHS) costs were also determined. Data were analysed by deprivation categories, using the Scottish Indices of Deprivation (2003), and by settlement type ranging from urban to very remote rural.

**Results:**

Uptake of screening was high in all settlement types (mean 89.6%, range 87.4 – 92.6%). Non-attendees were more deprived in terms of income, employment, education and health but there was no significant difference between non-attendees and attendees in terms of geographical access to services. Age was similar in both groups. The highest private costs (median £7.29 per man) and NHS screening costs (£18.27 per man invited) were observed in very remote rural areas. Corresponding values for all subjects were: private cost £4.34 and NHS cost £15.72 per man invited.

**Conclusion:**

Uptake of screening for abdominal aortic aneurysm in this remote and rural setting was high in comparison with previous studies, and this applied across all settlement types. Geographical location did not affect uptake, most likely due to the outreach approach adopted. Private and NHS costs were highest in very remote settings but still compared favourably with other published studies.

## Background

Screening for abdominal aortic aneurysm (AAA) has been carried out in the UK for over twenty years. The effectiveness of this intervention in detection and management of abdominal aortic aneurysms has been the subject of debate in the literature [1-5]. However, recent evidence suggests that screening for AAA can be clinically and cost effective in reducing aneurysm mortality among men aged 65–74 years [6-8].

The effectiveness of screening programmes has tended to be measured by the level of compliance or uptake, higher compliance equating with higher detection [9-11]. Although this approach has been questioned [12], it remains a key aspect in evaluations, particularly in the period prior to establishing national screening programmes. A systematic review of factors associated with uptake in various screening programmes identified a number of socio-demographic, attitudinal, social and health factors which influence compliance [13]. In that review, effects of geographical location on uptake were inconclusive. Geographical location of subjects and screening centres affects access to transport, distance to travel and costs of attending, including out-of-pocket expenses and the opportunity cost of time attending screening, i.e. time that could have been spent undertaking another activity.

Recent studies have suggested that private costs ought to be considered in screening evaluations [10,14-16]. Evidence on the impact of cost on uptake has tended to originate from the United States where screening incurs a charge [13]. While screening in the United Kingdom is typically offered free of charge, this overlooks the potentially considerable cost to the individual and the wider societal cost, for example through loss of productivity and care of dependants [10].

The cost-effectiveness of screening has generally been considered from a health care provider perspective, as in the recent UK multi-centre aneurysm screening study (MASS)[8]. Recognising the direct and indirect financial costs incurred by those invited to participate in a screening programme is beginning to form part of the evaluation process [10,17,18]. This is particularly relevant in remote and rural areas where access to screening may be limited by the availability of public transport and prohibitive costs associated with travel to screening centres [5,14,19].

Evidence suggests that rural residents face barriers in access to preventive health measures in comparison with urban residents, and, as a result, uptake has been typically low in rural areas [20]. This study considers the costs incurred by men participating in an AAA screening programme in the Highlands and Western Isles – a remote and rural area of the UK. In an attempt to overcome potential effects of rurality on uptake, screening was offered at many sites throughout the region. Potential differences arising from geographical location, and the associated private costs incurred, were explored in relation to the uptake of screening.

## Methods

The Highland Aneurysm Screening Programme (HASP) was established in 2001 to offer men in Highland and Western Isles aged 65–74 years screening for abdominal aortic aneurysm. Men born between 1927 and 1936, and currently registered with general practitioners (GPs) in Highland and Western Isles, were identified from Practitioner Services Division. GPs were asked to review lists and remove men who were no longer patients either due to death or moving away, or for whom an invitation to screening would be inappropriate due to known co-morbidity or previous aneurysm surgery. Invitations to attend a local screening session were sent out with an information leaflet and the opportunity to rearrange appointment time and date. One further invitation was sent to non-attendees. GPs were notified of patients who did not attend, and the file on each non-attendee was closed at that time.

A sonographer and screening nurse using a portable scanner carried out the screening sessions, at urban and community hospitals and general practice premises. Screening was offered at fifty-one locations across Highland and the Western Isles between February 2001 and January 2004. The land area of Highland (26,484 square kilometres) represents one third of Scotland and has a population of just over 208,000: 8 persons per square kilometre. The Western Isles, comprising sixty-six islands, many uninhabited, has a population of approximately 26,500 covering an area of 3,000 square kilometres.

Each man attending screening was asked to complete a questionnaire, providing information on the method of transport used to travel to the session; the duration of the journey; miles travelled by car users and costs incurred from public transport or taxi use. In addition they were asked whether they had taken time off work; whether wages had been lost as a result of attending; if they had been accompanied to the screening session and whether dependants had to be cared for to enable them to attend.

Direct costs to the individual were identified as out-of-pocket expenses arising from attending the screening session. The direct cost of travel was based on the actual cost of the return journey for those travelling by public transport or taxi. The cost of car travel was calculated at 45 pence per mile [21]. Wages lost calculations were based on information provided by participants or estimated using New Earnings Survey rates [22].

Indirect costs refer to the activity or opportunity foregone as a consequence of attending screening. In this study, the opportunity cost was calculated for men attending screening, their companion, if accompanied, and carer of dependants, where relevant. The rate was estimated at £3.79 per hour and was based on travel time [10,23]. Time spent at the screening session was not included. This rate was also applied to those who accompanied men to the screening session and the carer's time, where relevant. All financial estimates were based on financial year 2002/2003.

Remoteness was classified using the Scottish Household Survey (SHoS) eight-fold classification of settlements [24]. This classification, based on settlement size and drive times, was applied to the general practice at which each man was listed and to the screening sites. Using population data from the 2001 Census and Scottish Ambulance Service estimated drive time, each location was assigned a SHoS category. Postcodes of men invited to attend for screening were linked to the Scottish Indices of Deprivation 2003 [25], which are based on electoral wards. As well as providing an overall Index of Multiple Deprivation (SIMD), this has five domains, including income, employment, education, health, and geographical access to services.

NHS costs were calculated on the basis of prices for the 2002–3 financial year, and included administrative and staff cost as well as cost of equipment, as previously described [26]. We assumed an opportunity cost of zero for using NHS premises for screening, although our financial estimates could be altered to include a fee per session. Differences between screening locations reflected the variation in travel expenses and costs of travel time for the screening staff to attend each site. Central costs associated with administration of the programme and the costs of staff time for the screening session itself were assigned the same rates for all screening locations.

Data analysis was performed in SPSS version 14^®^. Mann-Whitney tests and unpaired t-tests were used to compare characteristics of non-attendees and attendees. Differences between settlement categories in respect of travel time, distance travelled and patient costs were explored using Kruskal-Wallis tests.

The Highland Research Ethics Committee approved the programme.

## Results

### Study sample

The Practitioner Services Division identified 9657 men in the appropriate age group. After excluding those who died before an appointment could be sent, or were no longer in the practice area or not at the address given, or were deemed inappropriate on grounds of co-morbidity or previous aneurysm surgery, 9323 were invited. For analysis purposes, useable postcodes for linkage to SIMD scores were available from 8542 (92%). Other postcodes were either missing or incomplete, or could not be matched to an electoral ward. Of those men invited, 8355 (89.6%) attended between April 2001 and January 2004 and 8,292 provided questionnaires.

The clinical outcomes of the programme have been published in detail elsewhere [26]. Uptake was high in all settlement types: urban 89.6%; accessible small town 87.9%; remote small town 87.4%; very remote small town 88.7%; accessible rural area 92.6%; remote rural area 92%; very remote rural area 88.9%. Of men screened, 430 (5.1%) had aortic diameter greater than 30 mm, indicative of aneurysm, and 40 (0.5%) had aortic diameter greater than 54 mm, the level at which surgery would be offered.

Characteristics of attendees and non-attendees are shown in table 1. Non-attendees were slightly more deprived on the overall Index of Multiple Deprivation (SIMD) and on most of the domains (Income, Employment, Education and Health). However, there was no significant difference between non-attendees and attendees on the Geographical Access to Services domain. Non-attendees were older than attendees, the mean difference being 2.4 months. However, since age was calculated for non-attendees at the date the file was closed, i.e. after a repeat appointment had been sent, but for attendees at the date of attendance, this difference probably reflects the time necessary to send a second appointment and close the file.

### Travel – Mode of transport

The method of travel used by men to attend screening, categorised by the location where screening took place, is shown in Table 2. Most men in all location categories travelled by car (83%). One in ten men walked to the session. The relative lack of availability of public transport is reflected in the small number of men using bus (5%) and train travel (0.1%). Significant differences were noted across the locations (X^2 ^= 315.9, p < 0.005). The greatest proportion of men travelling by bus was in urban areas, while the greatest proportion travelling by car was in very remote rural areas.

### Travel – Time travelled

Table 3 shows median journey times across the location categories. Significant differences in median journey time (X^2 ^= 257.6, p < 0.005) were noted. Men screened in urban and very remote rural areas spent more time travelling than men in other locations.

### Work/Activity foregone

A small proportion of men took time off work to attend screening (n = 325; 4%). Men no longer in employment had typically given up leisure pursuits to attend (67%).

### Companions and dependants

The majority of men attended screening alone (79%). 16% attended with a partner, 4% with a friend. The majority of companions had given up housework to accompany men (62%). Few men reported having to arrange for dependants to be looked after to enable them to attend screening (5%). Carers most frequently had foregone housework to look after dependants (48%).

### Costs

The private and NHS costs of the screening programme are shown in Table 3. The components of the private cost incurred by an individual attending screening were: travel, companion travel, work or activity foregone and care of dependants. Overall, the median private cost was £4.34. Private costs differed by location (X^2 ^= 136.5, p < 0.005). Men attending screening in very remote rural locations incurred the greatest private cost. NHS costs per session and per man screened were highest in the remote and very remote rural locations, reflecting the travel time and travel costs incurred by staff.

## Discussion

In this study, carried out in a rural and remote region of the United Kingdom, overall uptake of screening for AAA was 89.6%. This is higher than previous UK and European AAA screening studies (MASS 80%[7];Gloucester 84%[27]; Viborg, Denmark 76%[28]; Nijmegen, Netherlands 83%[29]). Non-attendees were more deprived than attendees, but geographical location of residence of those invited did not affect attendance. Private costs of attendance were highest for those screened in the most remote locations, as were NHS costs.

The high level of uptake in remote and rural locations contrasts with literature on the impact of rurality on access to preventive health services [30-33]. In an urban/rural comparison in the USA it was noted that after controlling for age, income, gender, education, race and ethnicity, rural residents remained less likely to access preventive health services [19]. The authors acknowledged that rural areas present particular challenges in improving access to preventive services and identify distance to travel and associated costs as an important factor. In addition poor quality roads and higher fuel costs were cited as an explanation for rural/urban differences in access to health care in Australia [33]. However, research in a rural area of Michigan concluded that travel time and distance were not associated with breast screening compliance, although the authors acknowledged that further research was required in a more remote area to explore the issue further [34]. In the current study, we believe that the potential negative effect of rural residence on uptake was overcome by taking screening sessions to as local a level as possible, primarily general practice premises.

The remote and rural nature of Highland and Western Isles meant that in many of the locations public transport was either not available or so limited that it was not a feasible option for attending screening. This was reflected in greater car use in the very remote rural areas. Time spent travelling was greatest for men living in the very remote rural and urban categories. This reflects the more distributed nature of very remote rural populations. In contrast, high travel times for those screened in urban areas are likely to reflect greater use of public transport and greater traffic congestion.

In a previous comparison of attendees and non-attendees in a remote rural mobile breast screening programme, it was found that non-attendees lived a greater distance from the mobile screening unit than participants. Keeping of appointment times was closely associated with the availability of public transport and those with appointments in the afternoon had the most difficulty in attending due to the absence of public transport at that time of day [14]. In our study, less than 5% of subjects travelled to screening appointments by public transport, and uptake of screening did not relate to the geographical access score of subjects' residences.

A review of factors that influence uptake of screening concluded that cost could not reliably be considered within the United Kingdom setting, in contrast with the United States where screening incurs a charge [13]. However, research in the UK has suggested that high private costs may act as a disincentive to attend preventive screening [10,15]. The evidence on the impact of private costs remains inconclusive. A study of patient costs in a hospital based AAA screening programme in Denmark found there was no correlation between costs to the individual and attendance rates [17]. However, in that study, opportunity foregone costs were calculated only for men in employment and therefore did not include the time costs of companions or those who had retired. That approach assigned no value to the time or activities given up by those who were not in employment. In our study, only a small number of men took time off work to attend screening (n = 325). The predominance of retired men had a considerable impact on the opportunity cost of time, reducing both the potential cost to the individual in terms of wages lost and the wider societal cost through lost productivity. In addition, less than a quarter of men (n = 1754) were accompanied to screening, lowering the wider societal cost. However, our finding that non-attendees had greater levels of material deprivation raises the possibility that cost may have influenced the decision to attend screening. Higher age and social deprivation were associated with poorer attendance at screening and follow up in the MASS trial [35].

In an economic evaluation of outreach assessment clinics for breast screening on three Scottish islands, higher health service costs were incurred through the cost of staff travel time and less efficient use of staff time. However this was set against reduced time and travel costs of women attending [36]. In our study, NHS costs were higher in the most remote locations, as were private costs, although the magnitude of excess private costs was relatively small, approximately £3 per subject above the average. In our study, it appears that potential barriers associated with time and travel were reduced and access was enabled through the outreach approach adopted. If screening had been restricted to the urban hospital base, considerable journeys would have been required for many of those participating.

There are some limitations of the present study. We were unable to link home postcodes to deprivation scores for 8% of our subjects. However, we do not believe that this would impact on our conclusions as the unmatched postcodes were equally distributed across all settlement types as determined from the location of the subjects' general practitioner premises. Our study was not designed to assess cost-effectiveness directly. However we observed similar clinical outcomes and NHS costs to the MASS trial [7] and thus the estimate from the economic evaluation of that trial [8] is a reasonable guide to cost-effectiveness in our study. The total NHS screening cost per man randomised for screening in the MASS study was £23.23, compared to £15.72 in our study. Thus even the addition of the private costs for our subjects result in a similar cost per man offered screening. It could be argued that the MASS trial findings imply that AAA screening is not cost-effective given a four- year time horizon. However, given a lifetime perspective screening it is almost certainly cost-effective.

## Conclusion

In conclusion, uptake of screening for abdominal aortic aneurysm in this remote and rural setting was high in comparison with previous studies, and this applied across all settlement types. Geographical location did not affect uptake, most likely due to the outreach approach adopted. Private costs and NHS costs were highest in very remote rural settings but still compared favourably with published data.

## Competing interests

The author(s) declare that they have no competing interests.

## Authors' contributions

SML collected and analysed the data and drafted the manuscript. JLD participated in the study design, data collection and analysis and drafting of the manuscript. JC participated in the study design, analysis and drafting of the manuscript. DJG participated in the study design, data analysis and drafting of the manuscript. All authors read and approved the final manuscript.

## Pre-publication history

The pre-publication history for this paper can be accessed here:



## Figures and Tables

**Table 1:** Characteristics of Non-attendees and Attendees

Variable	Non-attendees	Attendees	Significance
N	844	7968	
SIMD Score*	19.83 (11.35)	17.27 (11.8)	< 0.0001
Income Domain Score*	13.33 (7.34)	12.38 (6.76)	< 0.0001
Employment Domain Score*	13.64 (6.97)	12.38 (6.80)	< 0.0001
Education Domain Score*	-0.15 (0.86)	-0.30 (0.82)	< 0.0001
Health Domain Score*	-0.13 (0.69)	-0.26 (0.71)	< 0.0001
Access Score*	1.01 (1.83)	0.96 (1.73)	0.832
Mean (SD) Age*** (Years)	70.09 (2.93)	69.88 (2.88)	0.047

SIMD is the Scottish Index of Multiple Derivation composite score. All scores are shown as median (IQR) *Higher values indicate more deprived. **Age in years at date of file closure for non-attendees and date of attendance for attendees.

**Table 2:** Mode of travel to screening by screening location

Settlement Category	Car	Taxi	Bus	Train	Foot	Other
Urban*	1548 (77)	38 (2)	204(10)	2(0.1)	138 (7)	72 (4)
Accessible small town	606 (85)	8 (1)	23 (3)	1 (0.1)	74 (10)	4 (0.6)
Remote small town	221 (79)	2 (0.7)	6 (2)	0	49 (18)	1 (0.4)
Very remote small town	1685 (83)	49 (2)	97 (5)	4 (0.2)	181 (9)	14 (0.7)
Accessible rural	630 (83)	0	14 (2)	0	101 (13)	9 (1)
Remote rural	1012 (85)	4 (0.3)	15 (1)	0	136 (11)	21 (2)
Very remote rural	1156 (87)	11 (0.8)	36 (3)	1 (0.1)	102 (8)	17 (1)
All Settlements	6858 (83)	112 (1)	395 (5)	8 (0.1)	781 (9)	138 (2)

Data are n (%) *Urban, 10,000 –125,000 inhabitants. Classification of other areas based on settlement size and drive time to settlement of more than 10,000 inhabitants: Small town, 3,000–10,000; Rural, less than 3,000; Accessible, within 30 minutes drive; Remote, 30–60 minutes drive; Very remote, more than 60 minutes drive.

**Table 3:** Travel time, private cost and NHS cost of attending screening by screening location

Settlement Category	Travel Time (round trip) minutes Median (IQR)	Private Cost (£) Median (IQR)	NHS cost per session (£)	NHS cost per man invited (£)
Urban	30 (20)	4.2 (4.05)	248.79	14.34
				
Accessible small town	20 (20)	4.00 (4.86)	286.43	14.58
Remote small town	20 (20)	4.86 (7.83)	294.96	15.99
Very remote small town	20 (20)	3.96 (7.94)	274.65	14.56
Accessible rural	20 (20)	3.96 (5.76)	307.38	15.66
				
Remote rural	20 (30)	4.59 (8.87)	305.04	16.77
				
Very remote rural	30 (20)	7.29 (10.53)	332.66	18.27
All settlements	20 (30)	4.34 (6.85)	288.44	15.72
